# Anthocyanin-rich Aronia melanocarpa extract improves body temperature maintenance in healthy women with a cold constitution

**DOI:** 10.1186/2193-1801-2-626

**Published:** 2013-11-21

**Authors:** Keisuke Sonoda, Wataru Aoi, Tomoaki Iwata, Yanmei Li

**Affiliations:** Laboratory of Health Science, Graduate School of Life and Environmental Sciences, Kyoto Prefectural University, 1-5 Hangi-cho Shimogamo, Sakyo-ku, Kyoto, 606-8522 Japan; BGG Japan Co., Ltd, 8F YN Ginza Building, 8-14-11 Tokyo, Chuo-ku, Tokyo, Japan; Beijing Gingko Group Co., Ltd, 3F-KeHaiFulin Building, The Chinese Academy of Agricultural Sciences, No.12, Zhongguancun South Avenue, Beijing, Haidian District China

**Keywords:** Anthocyanin, Thermogenesis, Body surface temperature, Blood flow, Cold feeling

## Abstract

**Purpose:**

Specific anthocyanin-rich dietary factors have been shown to improve metabolic functions associated with thermogenesis in animal studies. *Aronia melanocarpa*, commonly known as wild chokeberry, contains a high level of anthocyanin that would be expected to maintain body temperature through thermogenesis. We here investigated the effects of *Aronia melanocarpa* extracts on body temperature and peripheral blood flow in healthy women with a cold constitution.

**Methods:**

A pre/post comparison trial was performed in 11 women with a cold constitution, who were taking *Aronia melanocarpa* extracts (150 mg/day) for 4 weeks. Physiological and biochemical parameters, along with psychological tests were examined.

**Results:**

The subjects’ body surface temperature was significantly higher in the post-trial than in the pre-trial. In psychological tests, factors related to cold were significantly improved by *Aronia* intake. On the other hand, peripheral blood flow was not affected by *Aronia* supplementation. Plasma noradrenalin level was significantly elevated by *Aronia* intake, and subjects with a higher level of 8-hydroxy-2'-deoxyguanosine in the pre-trial showed decreased levels in the post-trial.

**Conclusions:**

These data suggest that dietary *Aronia melanocarpa* extract improves the maintenance of body temperature in healthy women with a cold constitution, which may be mediated by noradrenalin and oxidative stress levels.

## Background

Peripheral cold constitution is a popular symptom among women and causes various health disorders such as shoulder stiffness, headache, swelling, sleeplessness, frequent urination, peripheral numbness, limb pain, chilblains, and purple fingernails (Melby 2007;Masuda et al. 2011). Body temperature is maintained by thermogenesis and thermal radiation. Thus, a reduction in thermogenesis leads to a cold feeling. In addition, it is thought that disturbances in peripheral circulation, induced by the contraction of peripheral vessels, play a role in thermal dysregulation. The contraction of peripheral vessels can be caused by sympathetic nerve activation, a decrease in endothelium-derived relaxant factor, or an increase in vasoconstrictor levels (Matz et al. 2000;Shepherd 1990). Therefore, if thermogenesis and peripheral circulation can be improved, the cold feeling may be alleviated.

Anthocyanins are the largest group of water-soluble pigments in the plant kingdom and are abundant in fruits, vegetables, beans, and red wine. Dietary anthocyanins have been shown to play an important role in the prevention of life-related common diseases, mainly as a result of their strong antioxidant and anti-inflammatory effects (Desjardins et al. 2012;Cuevas-Rodríguez et al. 2010). Anthocyanins improve the peripheral circulatory system by lowering blood pressure, maintaining the proper permeability and elasticity of arterial vessels, and activate nitric oxide (NO) to promote endothelial vasodilation (Jennings et al. 2012;Ojeda et al. 2010;Côrtes et al. 2002). In addition, several animal studies have shown that specific anthocyanins improve metabolism-associated thermogenesis in peripheral organs (DeFuria et al. 2009;Takikawa et al. 2010). Therefore, such dietary factors may be effective in improving cold constitution in humans.

Fruits berries are a dietary source of anthocyanin as well as many other essential nutritional components. *Aronia melanocarpa* is especially rich in polyphenols, in particular, anthocyanins as well as procyanidins and flavonoids (Kokotkiewicz et al. 2010). Previously, it has been shown that the intake of *Aronia* extract improves vascular function and metabolic activity along with antioxidant and anti-inflammatory (Broncel et al. 2010;Qin et al. 2012;Zapolska-Downar et al. 2012), which may contribute to the improvement of thermogenesis and peripheral circulation. Therefore, the aim of our study was to investigate the effects of *Aronia melanocarpa* extract supplementation on peripheral body temperature and blood flow in healthy women with a cold constitution.

## Results

### Body surface temperature and peripheral blood flow

**Figure 1:**
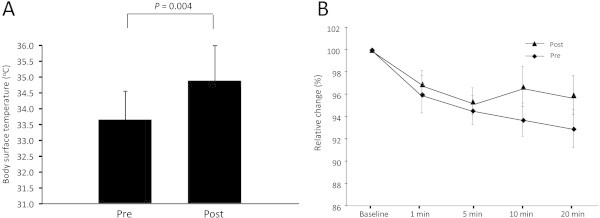
**Body surface temperature and peripheral blood flow. (A)** The maximum value for body temperature after 20 min of acclimatization in an air-conditioned room was measured. **(B)** The blood flow was examined during immersing their right hand into cold water for 20 min in a time-course manner. Values represent the mean ± standard deviation obtained from 11 subjects. Pre, pre trial; Post, post trial.

The maximum value for body temperature after 20 min of acclimatization in an air-conditioned room was significantly higher in the post-trial compared with the pre-trial measurement (P = 0.004) (Figure 1A). The relative mean value of blood flow in the subjects’ hands was calculated. The values were significantly decreased by cold water immersion over time in both the pre- and post-trials (Figure 1B). It showed a tendency towards suppression by 4 weeks of *Aronia* supplementation, but not significantly affected.

### Blood and urine parameters

**Figure 2:**
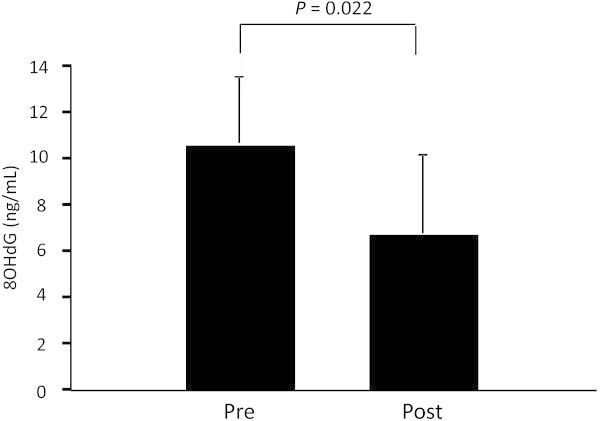
**Urine 8OHdG. Urine 8OHdG levels were measured in subjects who had baseline 8OHdG levels above 5 ng/mL.** Values represent the mean ± standard deviation obtained from 5 subjects. Pre, pre trial; Post, post trial.

The concentration of plasma noradrenalin significantly increased in the post-trial compared with the pre-trial (P = 0.027) (Table 1). In contrast, no significant changes were detected in plasma adrenalin, dopamine, nitrite/nitrate or cyclic guanosine monophosphate (cGMP) concentrations between pre- and post-trials. No significant change was observed in urine 8-hydroxydeoxyguanosine (8OHdG) levels between trials, although it was significantly decreased from 10.7 ± 3.0 ng/mL to 6.7 ± 3.4 ng/mL by *Aronia* intake in 5 subjects who had baseline 8OHdG levels above 5 ng/mL (P = 0.022) (Figure 2).

**Table 1:** **Concentration of plasma and urine parameters**

	Pre	Post
Plasma		
Adrenalin (ng·mL^-1^)	0.025 ± 0.014	0.031 ± 0.018
Noradrenalin (ng·mL^-1^)	0.225 ± 0.070	0.298 ± 0.099 ^a^
Dopamine (ng·mL^-1^)	0.013 ± 0.009	0.013 ± 0.005
Cyclic GMP (ng·mL^-1^)	3.14 ± 1.04	3.24 ± 1.10
Nitrite (μmol·L^-1^)	1.00 ± 0.00	1.00 ± 0.00
Nitrate (μmol·L^-1^)	23.4 ± 8.1	27.2 ± 12.7
Urine		
8OHdg (ng mL^-1^)	6.25 ± 4.74	6.50 ± 3.70

The plasma levels of catecholamines, cyclic GMP, and nitrite/nitrate as well as the urinary level of 8OHdG were examined. Pre, pre-trial; Post, post-trial. ^a^, significant deference between the Pre at the level of *P* < 0.05.

### Psychological tests

The value related to a cold feeling in the hand, foot, and hip was significantly improved after supplementation compared with the baseline (Table 2). On the other hand, we did not observe any significant changes in the value for a cold feeling of the back and shoulder stiffness between trials.

**Table 2:** **Psychological test for the examination of subjective peripheral blood flow**

	Pre	Post
Cold feeling		
Hand and foot	3.18 ± 1.54	4.17 ± 0.84 ^a^
Hip	3.18 ± 1.54	4.23 ± 0.82 ^a^
Back	3.64 ± 1.21	4.26 ± 0.77
Shoulder stiffness	2.91 ± 1.45	3.43 ± 0.97

The subjects’ state of psychological stress was assessed using a questionnaire regarding cold feelings in the hand/foot, hip, and back as well as shoulder stiffness. The questionnaire consisted of 5 responses, namely, 1, feel very cold or stiffness; 2, feel somewhat cold or stiffness; 3, do not feel anything; 4, almost do not feel cold or stiffness at all. Values represent the mean ± standard the deviation obtained from 11 subjects. Pre, pre-trial; Post, post-trial. ^a^, significant difference from the Pre at the level of *P* < 0.05.

## Discussion

The present study revealed the following novel observations: (1) *Aronia* supplementation for 4 weeks elevated body surface temperature, (2) *Aronia* increased plasma noradrenalin concentration, and (3) *Aronia* attenuated the cold feeling in all 4 limbs and the hip, as determined by the VAS test, in 11 women with a cold constitution. Cold constitution causes various symptoms such as shoulder stiffness, headache, swelling, frequent urination, peripheral numbness, lumbar pain, and limb pain. Thus, the attenuation of the cold feeling in all 4 limbs and the hip by *Aronia* supplementation is associated with an improvement in cold constitution. Previously, it has been shown that several polyphenols can activate thermogenesis by improving the metabolism in metabolic organs and cells in animal and cell culture studies (Alberdi et al. 2013;Dulloo et al. 2000;Moon et al. 2013;Shixian et al. 2006); however, it has not been shown in humans. To the best of our knowledge, our observations are the first to demonstrate that polyphenol-rich food extracts improve a cold constitution in human subjects through thermoregulation and redox regulation.

*Aronia melanocarpa* contains polyphenols such as anthocyanins, procyanidins, and flavonoids that can improve nutrient metabolism and possess antioxidant and anti-inflammatory properties. Previously, it has been shown that some factors elevate thermogenesis in peripheral metabolic organs. Takikawa et al. (2010) reported that an anthocyanin, cyanidin 3-glucoside, enhances adiponectin secretion and upregulates the expression of thermogenesis-related proteins, such as uncoupling proteins, in isolated rat adipocytes. Cyanidin 3-glucoside also activates adenosine monophosphate (AMP)-activated protein kinase (AMPK) in skeletal muscle and liver. In addition, quercetin-rich onion peel extract elevates uncoupling protein-1 expression and inhibits adipogenesis in both 3 T3-L1 cells and in rats fed high-fat diets (Moon et al. 2013). In addition to the direct effect of anthocyanins on improving metabolism in metabolic organs, circulating levels of catecholamine may be an inducer of thermogenesis in these organs. It is well known that noradrenaline elevates thermogenesis *via* cyclic AMP and uncoupling proteins after binding to the beta-adrenalic receptor in adipose and skeletal muscle tissues (Lowell et al. 2000;Rehnmark et al. 1990). We found increased levels of plasma noradrenalin after 4 weeks of *Aronia* intake, which may contribute to the attenuation of cold feeling through thermogenesis. On the other hand, autonomic nerve regulation may be associated with elevated noradrenalin, although it remains unclear. AMPK can be activated by some anthocyanins and is closely associated with sympathetic nervous system regulation. Several studies have shown that central AMPK stimulates sympathetic nerve activity that can induce the secretion of noradrenalin (Tanida et al. 2011;Viollet et al. 2003).

Reactive oxygen species generated in the vascular endothelium can then react with NO·, reducing its vasoactive levels (Förstermann 2010). This diminishes the response to endothelium-dependent vasodilators via the formation of peroxynitrite anions (ONOO^-^), a highly reactive intermediate with strong cytotoxic potency. Thus, antioxidant factors can promote a vasodilator effect of NO by scavenging reactive oxygen species. In fact, the intake of dietary antioxidants including vitamins, carotenoids, and polyphenols has been reported to show vasodilation action (Barona et al. 2012;Pleiner et al. 2008;Zhao et al. 2011). A number of studies have shown that flavonoids are superior to vitamins E and C as antioxidant agents. In addition, certain polyphenols have been observed to reduce the generation of reactive oxygen species from the endothelium or phagocytes (Cai et al. 1997), which can also contribute to decreasing the oxidative stress level. *Aronia* has been shown to attenuate exercise-induced oxidative damage associated with an improvement in exercise tolerance and other physiological parameters (Pilaczynska-Szczesniak et al. 2005). In the present study, dietary *Aronia* extracts caused a reduction in urine 8OHdG levels in subjects with a higher oxidative stress level before the trial. This may result in endothelium vasodilation by reducing oxidative stress. On the other hand, noradrenalin functions as an endothelium vasoconstrictor in the peripheral vein. Thus, the increase of noradrenalin by *Aronia* intake may lead to contraction of the peripheral vein. Nevertheless, blood flow was not decreased by *Aronia* intake, which suggests that vasodilation induced by reducing oxidative stress may compensate for the vasocontraction in response to noradrenalin, leading to an improvement in the cold feeling.

## Conclusion

Body surface temperature and plasma noradrenalin levels were significantly elevated by *Aronia* supplementation. In psychological tests, factors related to cold were significantly decreased by *Aronia* intake. Subjects with higher 8OHdG levels at baseline showed decreased levels in the post-trial period. These data suggest that dietary *Aronia melanocarpa* extracts improve the maintenance of body temperature in healthy women with a cold constitution, which may be mediated by noradrenalin and oxidative stress levels. On the basis of the results of this pilot study, further research is required in a placebo-controlled study to determine whether these findings can be generalized to a larger population.

## Methods

### Subjects

Twelve healthy Japanese women, aware of their cold constitution, were recruited for this study using the following inclusion criteria: 40–60 years of age, normal menstrual cycle and dietary habits, and no severe physical or psychological illnesses. A pre-examination was performed to objectively determine the degree of the women’s cold constitution. Subjects with severe cold constitution were screened by performing a pre-test where subjects immersed their right hand into cold water for 20 min and subsequently, the blood flow was determined. Ultimately, 11 women (33.2 ± 4.9 yr) who were judged to have high cold constitution were asked to participate in this study and underwent a medical examination. The examination included an interview with a physician and blood and urine analyses before commencement of the study. All subjects were free of signs, symptoms, and history of any overt chronic disease. This study was approved by the ethics committee of Shiba Palace Clinic and all subjects signed a consent form after reading the study protocol.

### Aronia tablet

Frozen *Aronia melanocarps* fruits were extracted by ethanol/water, followed by chromatography and spray drying. The whole process was performed under low temperature and with nitrogen protection, so that the anthocyanins may not be oxidized during the purification. *Aronia* tablets were comprised after added calcium stearate and silicon dioxide. Each tablet (250 mg) included *Aronia melanocarpa* extract (50 mg), crystalline cellulose (25 mg), and starch hydrolysate (162.5 mg), calcium stearate (7.5 mg), and silicon dioxide (5 mg). The content of anthocyanin contained in *Aronia melanocarpa* extract was equivalent to 35% w/w.

### Study design

A pre/post comparison trial was performed. Body surface temperature, peripheral blood flow, blood and urine biochemical parameters, and psychological tests related to cold were measured for baseline measurements. All subjects took 3 *Aronia* tablets, daily, with water 30 min after breakfast for 4 weeks. The measurements were taken the day after the final tablet ingestion. Subjects were asked to refrain from consuming caffeine and alcohol, and intense physical activity 24 h before each trial.

### Body surface temperature and peripheral blood flow

Peripheral body temperature was analyzed by thermography (Neo Thermo TVS-700, Nippon Avionics Co., Ltd., Tokyo, Japan). Subjects were acclimatized to an air-conditioned (25 ± 1°C/50 ± 10% room humidity) room for 20 min and the temperature of their right hand was measured using thermography. The point of maximum temperature in the thermogram image was examined.

Blood flow on the tip of the middle finger was measured using a laser Doppler flowmeter (PMEGAZONE OZ-1, OMEGAWAVE Ltd, Tokyo, Japan). Subjects immersed their right hand into cold water for 20 min and the blood flow was examined in a time-course manner.

### Blood and urine parameters

Subjects provided a urine specimen from an overnight accumulation, returned to the laboratory at 9:00 am while maintaining their fast, sat on a chair, and were made to rest. Subsequently, blood was collected from the antecubital vein. Immediately after collection, each blood sample was centrifuged and plasma was obtained. The plasma levels of catecholamines (adrenalin, noradrenalin, and dopamine) and nitrite/nitrate were measured using high performance liquid chromatography. Plasma cGMP levels were measured by radioimmunoassay (YAMASA Co., Ltd., Chiba, Japan). The urine concentration of 8OHdG, a marker of deoxyribonucleic acid oxidative damage, was measured using an enzyme-linked immunsorbent assay kit (Japan Institute for the Control of Ageing, Fukuroi City, Shizuoka, Japan).

### Psychological tests

The subjects’ state of psychological stress was assessed using a questionnaire. The questionnaire consisted of 5 responses, namely, 1, feel very cold; 2, feel somewhat cold; 3, do not feel anything; 4, almost do not feel cold; 5, do not feel cold at all, for questions regarding cold feelings in the hand/foot, hip, and back. The subjects selected their perception of the current state of their hand, foot, hip, and back from the 5 sections. Similarly, a questionnaire was used to examine the degree of shoulder stiffness.

### Statistical analyses

The differences between pre- and post-experiment parameters were evaluated by the Wilcoxon signed-rank test. Statistical significance was accepted at P < 0.05. All data are reported as the mean ± standard deviation.
